# The Effect of Being Pregnant during Respiratory Pandemics: A Comparison between 2009/10 Flu and 2020/21 COVID-19 Pandemic in Brazil

**DOI:** 10.3390/vaccines10081202

**Published:** 2022-07-28

**Authors:** Ana Beatrice Bonganha Zanon, Elias Ribeiro Rosa Júnior, Nátaly Adriana Jiménez Monroy, Luciana Graziela de Godoi, Bruna Rodrigues de Mattos, Cristiane de Freitas Paganoti, Rossana Pulcineli Vieira Francisco, Agatha Sacramento Rodrigues, Rafaela Alkmin da Costa

**Affiliations:** 1 Divisão de Clinica Obstetrica, Hospital das Clinicas HCFMUSP, Faculdade de Medicina da Universidade de São Paulo, 255 Dr. Eneas Carvalho de Aguiar Avenue, 10th Floor, São Paulo 05403-000, Brazil; ana.zanon@fm.usp.br (A.B.B.Z.); c.paganotti@hc.fm.usp.br (C.d.F.P.); 2 Departamento de Estatística, Universidade Federal do Espírito Santo, 514 Fernando Ferrari Avenue, Goiabeira, Vitória 29075-910, Brazil; elias.rosa@edu.ufes.br (E.R.R.J.); nataly.monroy@ufes.br (N.A.J.M.); luciana.godoi@ufes.br (L.G.d.G.); bruna.mattos@edu.ufes.br (B.R.d.M.); agatha.rodrigues@ufes.br (A.S.R.); 3 Disciplina de Obstetrícia, Departamento de Obstetrícia e Ginecologia, Hospital das Clinicas HCFMUSP, Faculdade de Medicina da Universidade de São Paulo, São Paulo 05403-000, Brazil; rossana.francisco@hc.fm.usp.br

**Keywords:** pregnancy, Influenza A virus, H1N1 subtype, COVID-19, mortality

## Abstract

Pregnant women undergo physiological changes that make them a challenging group of patients during pandemic respiratory diseases, as previously found during H1N1 2009 pandemic and recently ratified in COVID-19 pandemic. We conducted a retrospective cohort analysis on 5888 hospitalized women for H1N1 flu pandemic (2190 pregnant and 3698 non-pregnant) and 64,515 hospitalized women for COVID-19 pandemic (5151 pregnant and 59,364 non-pregnant), from the Brazilian national database, to compare demographic profile, clinical aspects, and mortality in childbearing aged women during both pandemics. Additionally, the effect of being pregnant was compared between both pandemics. In both pandemics, pregnant women were younger than non-pregnant women. Overall, pregnant women had lower frequencies of comorbidities and were less symptomatic. Among hospitalized women, pregnant women presented lower mortality rates than non-pregnant women (9.7% vs. 12.6%, *p* = 0.002 in the H1N1 pandemic and 9.7% vs. 17.4%, *p* < 0.001 in the COVID-19 pandemic) and this difference was statistically more pronounced in the COVID-19 pandemic, even after balancing pregnant and non-pregnant groups regarding age and chronic diseases.

## 1. Introduction

Pregnant women are a distinct group of patients during pandemics and are often considered at higher risk for morbidity and mortality than the general population. This is due to the changes that occur in the female body during pregnancy, such as immunosuppression, decreased lung expansion, cardiopulmonary adaptations to pregnancy, the worsening of pre-existing comorbidities, and the emergence of obstetric diseases. This makes pregnant women more susceptible to complications and death from infectious diseases [1,2].

In the pandemic caused by the influenza virus, which began in Brazil in April 2009, the severity and lethality in pregnant women (especially in the third trimester) was alarming—there was a 4-fold increase in indirect maternal deaths recorded in 2009 from respiratory diseases, complicated by pregnancy, childbirth, and puerperium [3]. This increase in maternal deaths did not only occur in Brazil; Callanghan et al., showed that, in the United States between 2009 and 2010, 12% of pregnancy-related deaths were attributed to confirmed or probable cases of Influenza A infection [4]. This rise in maternal deaths evidenced the need for public policies in order to assign priority and to facilitate the access of pregnant women to the health care services and proper treatment during outbreaks of influenza [5]. This concern about the pregnant population might partially explain why some Brazilian authors did not find a statistical difference of lethality between pregnant and non-pregnant women during the H1N1 2009 pandemic [6]. In fact, the concern about considering pregnant women as a vulnerable group and their prioritization to healthcare assistance may be some reasons why other authors might also have failed in finding differences in death rate between pregnant and non-pregnant women during the influenza pandemic [7,8].

Coronaviruses have been responsible for two important respiratory pandemics during the last decades (severe acute respiratory syndrome coronavirus—SARS-CoV—and the Middle East respiratory syndrome coronavirus—MERS-CoV), which evidenced pregnant women as a very vulnerable population, once nearly one third of the infected pregnant women died from severe acute respiratory syndrome (SARS) [9]. Nonetheless, at the beginning of the recent SARS-CoV-2 pandemia there was uncertainty if the disease would be more severe to the pregnant than to non-pregnant women, but, based on previous experiences of coronavirus and pregnancy, pregnant women should be considered as high risk for developing severe COVID-19 infection [10]. The first reports from China showed that the infection affected pregnant and non-pregnant women with a similar frequency and severity [11]. However, with the growing number of cases, an increase in complications and deaths in pregnant women was observed, especially in the last trimester and in the puerperium [12,13]. Thus, international health agencies, such as the World Health Organization and the Ministry of Health of Brazil, advised that pregnancy should be considered a risk factor for COVID-19, in addition to smoking, obesity, heart disease, hypertension, severe lung disease, immunosuppression, chronic kidney disease, diabetes mellitus, and neoplasia [14].

This way, pregnant women appeared once again as a special group to be focused on during the COVID-19 pandemic, especially in low- and middle-income countries [15].

Since in Brazil the H1N1 pandemic had massively affected pregnant women, highlighting them as a vulnerable population and determining that they should be addressed with special care in cases of H1N1 infection, COVID-19 infection in pregnant women raised concern about if this respiratory infection would have the same impact on this population and if healthcare policies should also be addressed to protect this vulnerable group.

The present study aims to evaluate the epidemiological and clinical data of hospitalized women of childbearing age and to compare pregnant and non-pregnant women regarding their demographic, clinical profiles, and death rate during the two pandemics (H1N1 and COVID-19), and then to compare the differences between these groups—in other words, to compares the odds ratios between pregnant and non-pregnant women—between the two pandemics.

## 2. Materials and Methods

### 2.1. Study Design and Population

We performed a retrospective cohort analysis of the information from the Sistema de Informação de Vigilância Epidemiológica da Influenza (SIVEP-Gripe) database. The SIVEP-Gripe is a Brazilian national database, created in 2000, to monitor virus circulation and respiratory infections across the country. Flu cases should be notified for individuals presenting fever and cough and/or sore throat in sentinel monitoring units (hospitals and local health departments) designated to register and report these cases. Virus surveillance for public health purposes has dynamic characteristics and frequent updates are made in notification guidelines, as was the case for the H1N1 and COVID-19 pandemics. The SIVEP-Gripe records contain demographic, clinical, and epidemiological data, as well as laboratory/etiological results. There is also information about hospital admission and disease progression (cure or death). The database allows public and free access by the following electronic addresses: https://opendatasus.saude.gov.br/dataset/bd-srag-2009-a-2012, accessed on 1 October 2021, https://opendatasus.saude.gov.br/dataset/bd-srag-2020, accessed on 1 October 2021, and https://opendatasus.saude.gov.br/dataset/bd-srag-2021, accessed on 1 October 2021.

In 2009, with the burden of the H1N1 pandemic, a rigorous surveillance of the cases of severe acute respiratory syndrome (SARS) was adopted, with compulsory notification of all SARS cases. The definition of SARS included the presence of fever, cough, and dyspnea. Since 2010, only hospitalized cases of SARS must be notified, both in public and in private hospitals; all cases of deaths caused by SARS, irrespective of hospitalization, should be notified.

At the time of COVID-19 pandemic, cases of SARS must be notified in the presence of at least two of the following symptoms: fever, chills, sore throat, headache, cough, runny nose, olfactory or taste disorders and dyspnea, chest pressure, saturation less than 95%, or blue coloration of lips or face. For COVID-19, only hospitalized cases of SARS must be notified and all SARS-related deaths must be notified.

Since SIVEP-Gripe is an open database, with no possibility of individual identification, according to Brazilian regulations of the National Research Ethics Commission (Comissão Nacional de Ética em Pesquisa—CONEP), this study does not require prior approval by the institutional ethics board [16].

### 2.2. Data Collection

For the present analysis, data from the first 13 months of each pandemic were retrieved from the database: from May 2009 to May 2010 for H1N1; and from March 2020 to March 2021 for COVID-19. The search included data from female patients of childbearing age (10 to 49 years) with positive RT-PCR diagnosis for H1N1 (Flu virus) and SARS-CoV-2 (COVID-19 virus).

Cases were excluded if they lacked information about the pregnancy status or if the PCR result was unavailable to confirm the final diagnosis.

To avoid the bias caused by the different notification criteria between both pandemics (and even within the H1N1 pandemic), we decided to select only hospitalized patients.

For each pandemic, childbearing aged females were divided according to the pregnancy status, into PREGNANT or NON-PREGNANT and compared regarding the following epidemiological characteristics: age, ethnicity, previous influenza vaccination, need to travel and change city to access health care, presence of comorbidities, disease presentations, symptoms, and main outcome (death or cure).

### 2.3. Statistical Analysis

The analyses were performed with the free-statistical software R (R Foundation for Statistical Computing Platform, version 4.0.3, Vienna, Austria, 2020. Available online: https://www.r-project.org/, accessed on 1 October 2021) [17].

Categorical data were compared by chi-squared test or Fisher exact test, when applicable. Continuous data were compared by *t*-Student test. Effect size of the differences between two groups in respect to continuous variables was assessed by d-Cohen measure [18]. Odds ratios (OR) and 95% confidence intervals (95% CI) were estimated for each comparison and statistical significance was assumed when *p* < 0.05.

The Breslow–Day test for homogeneity of OR was used in order to assess potential variations in the effect sizes for PREGNANT or NON-PREGNANT across the pandemics [19]. Thus, the Breslow–Day test was used to compare the protection/risk effect of being pregnant for each variable between the H1N1 flu and COVID-19 disease pandemics.

Since the disbalance between PREGNANT and NON-PREGNANT groups could be distinct in each pandemics and, therefore, cause bias in comparing the OR for death in pregnant women between the two pandemics, both PREGNANT or NON-PREGNANT groups were balanced (within each pandemic) regarding age and chronic diseases by applying the propensity score matching (PSM) technique [20]. PSM was carried out with the R Weightlt package considering logistic regression to create the weights and the average treatment effect (ATE) as the estimand method for treatment effects based on inverse probability of treatment weighting method (IPTW) [21,22].

## 3. Results

### 3.1. Study Population

**Figure 1:**
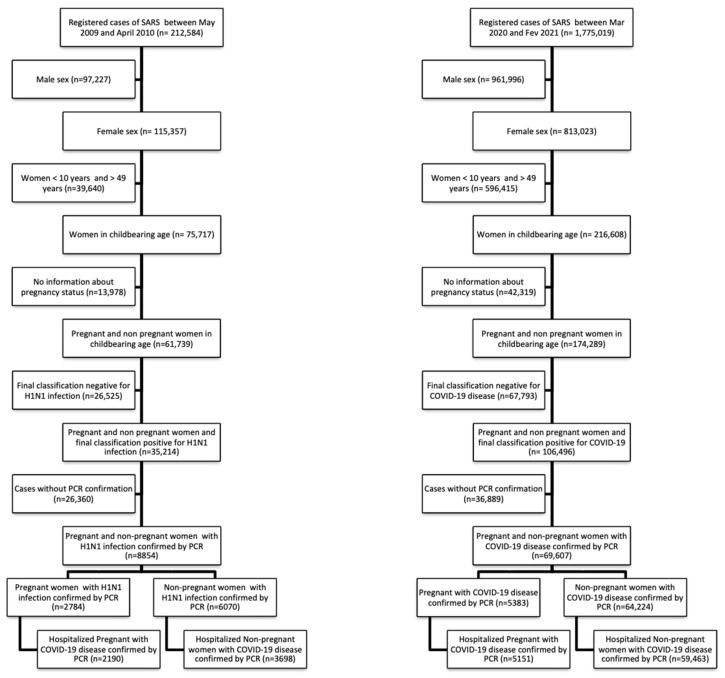
Selection of subjects included in the analysis for both H1N1 and COVID-19 pandemics in Brazil.

A total of 212,584 cases of SARS were reported within the first 13 months of the H1N1 flu pandemic and 1,775,019 cases of SARS during the first 13 months of the COVID-19 disease pandemic in Brazil. Among them, 5888 cases of H1N1 flu (2190 pregnant and 3698 non-pregnant) and 64,614 cases of COVID-19 disease (5151 pregnant 59,463 non-pregnant) were included in the analysis (Figure 1).

### 3.2. Baseline Characteristics of the Subjects Enrolled

Epidemiological and clinical characteristics of the population are presented in Table 1. During both pandemics, pregnant women were younger than non-pregnant women, but the effect size was smaller in the H1N1 pandemic (Cohen’s d = 0.27) than in the COVID-19 pandemic (Cohen’s d = 1.04). There was a clear predominance of white ethnicity among women infected with H1N1, both in pregnant (64.2%) and non-pregnant (77.1%) groups, in contrast to a higher frequency of brown ethnicity in subjects affected by COVID-19 disease, especially in pregnant group (47.9%).

Comorbidities were more frequent among non-pregnant women in both pandemics and that could be observed for almost all reported chronic diseases (cardiac, respiratory, renal, and immunosuppression). Hematologic diseases were more frequent among non-pregnant groups only in the COVID-19 pandemic. Nonetheless, comparing the odds ratios with the Breslow–Day test, the differences between pregnant and non-pregnant women were not statistically significant by contrasting the two pandemics. (Table 1).

During the COVID-19 pandemic, pregnant women had to travel more frequently to a city different from where they lived in to receive health assistance (36.5% vs. 28.8%, *p* < 0.001). This was different from what happened during the H1N1 pandemic, when health care access was not different between pregnant and non-pregnant subjects.

During the H1N1 pandemic, pregnant women had lower rates of flu vaccination than non-pregnant women (4.9% vs. 8.7%, *p* < 0.001). This scenario changed across the last decade; during the COVID-19 disease, previously flu-vaccinated pregnant women were almost double that of non-pregnant women (OR 1.94, 95% CI 1.78–2.12).

### 3.3. Clinical Manifestations and Outcomes of COVID-19

Clinical presentation of both groups is presented in Table 2, both for H1N1 and COVID-19 pandemics. Overall, pregnant women presented less symptoms than non-pregnant women, with less presentations of dyspnea, fever, sore throat, diarrhea, and cough (the last one only for COVID-19 pandemic). Nonetheless, when comparing the two pandemics, only the difference in the OR for fever and dyspnea was significant between the groups, being even lower in hospitalized pregnant women than in non-pregnant women during the COVID-19 pandemic (OR 0.71, 95% CI 0.62–0.80 vs. OR 0.49, 95% CI 0.46–0.52, *p* < 0.001 for the Breslow–Day test).

The death rate was lower among pregnant women than in non-pregnant women (Table 3) both during H1N1 disease (9.7% vs. 12.6%, *p* = 0.002) and during the COVID-19 disease (9.7% vs. 17.4%, *p* < 0.001). According to the Breslow–Day test, this protection effect of pregnancy was higher during the COVID-19 pandemic (OR 0.75; 95% CI 0.62–0.89 vs. OR 0.51; 95% CI 0.46–0.57, *p* < 0.001 for Breslow–Day test). In order to avoid potential bias for non-pregnant women being older and presenting higher frequencies of chronic diseases, pregnant and non-pregnant groups were balanced with each pandemic regarding age and chronic diseases with PSM. After balancing the groups, the differences between the OR for death in pregnant women remained significant (OR 0.80; 95% CI 0.71–0.91 for H1N1 vs. OR 0.66; 95% CI 0.64–0.68 for COVID-19, *p* = 0.02 for Breslow–Day test).

## 4. Discussion

We observed significant differences in the epidemiological characteristics between hospitalized pregnant and non-pregnant women in the H1N1 and COVID-19 pandemics. Pregnant women were younger, had a lower frequency of comorbidities, and were less symptomatic than non-pregnant women. However, comparing H1N1 and COVID-19 pandemics, the difference between groups presented statistical significance only regarding age, fever, and dyspnea. In the COVID-19 pandemic, pregnant women more frequently had to travel to receive health assistance. In both pandemics, hospitalized pregnant women presented lesser death rates than hospitalized non-pregnant women and, comparing H1N1 and COVID-19 pandemics, this difference was more pronounced during the last pandemic.

The mean age was significantly higher in the non-pregnant group for both diseases. However, there was a larger effect size in COVID-19 than in the H1N1 pandemic. Comparing pregnant women in H1N1 and COVID-19 pandemics, the mean age was 5 years higher in the COVID-19 pandemic. This may reflect a change in the profile of Brazilian pregnant women in the last 10 years, with a progressive increase in the number of pregnant women aged 35 years or older [23].

In both pandemics, non-pregnant women had a higher frequency of comorbidities than pregnant women but the differences of the OR were not statistically significant, meaning that in both pandemics the higher prevalence of chronic diseases in non-pregnant women were similar. As the mean age between the groups was around 8 years during the COVID-19 pandemic, it was expected that non-pregnant women would have more chronic diseases. On the other hand, during the H1N1 pandemic, the groups are more homogeneous regarding age, with a mean age difference of only 1 year between pregnant and non-pregnant women. Thus, the difference between ages does not justify a significant difference in the frequency of comorbidities. Perhaps, in the H1N1 pandemic, the presence of comorbidities was a more relevant factor to the non-pregnant women for the most severe and, therefore, hospitalized cases of disease, while pregnancy itself was a sufficient risk factor for H1N1 severity, even in the absence of comorbidities, given the similarity in the absolute difference of age between pregnant and non-pregnant women during this pandemic.

Ideally, pregnant and non-pregnant women should have access to the health system without having to travel to a different city. According to Leal et al., peregrination for delivery occurs in 23.5% of overall Brazilian pregnancies, irrespective of any epidemiological burden, which already calls attention to a basal deficiency in the Brazilian antenatal care system [24]. In the H1N1 pandemic, there was no significant difference between the groups in terms of need for displacement. However, during the COVID-19 pandemic, pregnant women had to travel 1.42 times more frequently from their cities to receive health care than non-pregnant women. Considering a decade has passed between pandemics, it was expected that access to the healthcare system would have increased for the COVID-19 pandemic, with less need for displacement. However, the opposite was observed and, perhaps, what can justify this fact is the greater number of people infected by coronavirus than by H1N1 in the general population, overcrowding hospitals. In addition, because pregnancy is considered a risk factor for the disease, these patients may have been more frequently referred to high-complexity maternity hospitals with specialized care in COVID-19, which are mostly concentrated in large cities, such as Brazilian state capitals.

Flu vaccination gained importance after the H1N1 pandemic. In April 2010, pregnant women became a priority group in the national immunization program, with vaccines available free of charge at health centers for all pregnant women, regardless of gestational age. Public campaigns to encourage vaccination and warning about pregnancy as a risk factor for influenza were responsible for greater adherence to vaccination [25]. There was an increase from 4.9% to 36.3% in vaccinated pregnant women among those who were hospitalized, when comparing the two pandemics. However, even with all these efforts, the vaccination rate is still low, with only 1/3 of hospitalized pregnant women with COVID-19 vaccinated against influenza. Unfortunately, this is not an exclusively Brazilian scenario; Lim et al., showed that in the United Kingdom, by December 2010, less than 50% of the young population at risk, including pregnant women, had been vaccinated [26]. In addition, recently, some data have shown a protection effect of influenza vaccines against poor outcomes for COVID-19 SARS [27], which should be an additional reason to reinforce and to stimulate the vaccination of pregnant women. One of the reasons for low adherence to vaccination is the fear for vaccine safety, even though it has already been proven. Thus, it is important that during prenatal care physicians recommend the vaccine, clarify doubts, reinforce safety and explain the importance of vaccination for maternal–fetal health.

Although the current knowledge about the protection effect of COVID-19 vaccination to pregnant women [28], during the period comprising this analysis, COVID-19 vaccines were not available to pregnant women in Brazil, neither this information was contemplated within the SIVEP-gripe form. Therefore, the effect of COVID-19 vaccination could not be further explored in this study.

Symptoms were less frequent in hospitalized pregnant women than in hospitalized non-pregnant women, both in H1N1 and in COVID-19 pandemics. This may reflect that the threshold for hospitalization could have been lower to pregnant women than for non-pregnant women, so that hospitalized pregnant women more frequently presented milder cases of the diseases. This is in agreement with the recommendations of public policies to assign priority to pregnant women during health calamities as an attempt to mitigate the devastating effect that respiratory pandemics can cause to this vulnerable population [5,15].

It is important to note that, at the beginning of the H1N1 pandemic, all suspected cases with clinical symptoms were reported; then, only cases with dyspnea and, later, only hospitalized cases. Thus, many—but not all—mild cases were initially reported, unlike what occurred in the COVID-19 pandemic, where only death and/or hospitalized cases were supposed to be reported. This difference in notification criteria justifies a lower rate of hospitalized cases during the H1N1 pandemic than in COVID-19 pandemic (overall, 66% of the H1N1 notified cases and 93% of the notified COVID-19 cases, as observed in Figure 1). Nonetheless, assuming that the notification criteria was the same for both pregnant and non-pregnant women for every considered pandemic moment, we should mention that among the notified cases, pregnant women were more likely to be hospitalized (80.4% vs. 63.1% for H1N1 and 96.9% vs. 94.6% for COVID-19 pandemic, *p* < 0.001 for both comparisons). Since pregnant women were less symptomatic and presented a lower death rate, we could hypothesize that the threshold to hospitalize pregnant women during both pandemics could have been lower, with milder cases being hospitalized. However, that should be considered with caution because, since there is no database about the non-hospitalized cases of flu-like diseases, we cannot analyze the real rate of hospitalizations in both pandemics.

In view of the aforementioned notification criteria bias, in order to better match the selection of cases among the pandemics, we chose to analyze and assess mortality only among hospitalized women. By selecting only hospitalized cases, we observed that mortality was lower in the group of pregnant women in both pandemics, with an even greater reduction in the group of pregnant women with COVID-19. Saraceni et al., reinforces this finding and did not report a higher mortality related to H1N1 infection in pregnant women than in non-pregnant women in the city of Rio de Janeiro in 2009, besides presenting a higher frequency of hospitalization among the pregnant group [6]; in their report, the intensive care unit admission rate was also higher among pregnant women. Although their findings did not reach statistical significance, possibly due to the small sample size, their results are in line with those published by Lim et al. [7] in Singapore and by Dolamn et al. [8] in the United Kingdom, who found higher risk of ICU admission and mechanical ventilation in pregnant than in non-pregnant women but failed to find significant difference in the death rate between groups. In the same way, Lenzi et al., also did not associate pregnancy with higher mortality [29] and the authors also referred to the prioritization of hospitalization and antiviral treatment as potential factors that might have influenced the fatality rate during the H1N1 pandemic. The increase in admissions was also observed in the United States, and Jamieson et al., reported that pregnant women were admitted to hospitals four times more than the general population [30].

A similar scenario was illustrated by Khan et al. [31] regarding COVID-19 pandemic. Pregnant women were less symptomatic at admission than non-pregnant women, but they were more frequently admitted at ICU and submitted to invasive mechanical ventilation. Nonetheless, no significant difference of death rate was found between groups.

Our results are in line with this data, since we also found pregnant women to present less symptomatic clinical presentation of both H1N1 and COVID-19 infection, but, in contrast, we found lower death rates in pregnant women than in non-pregnant women and this difference was even more pronounced in the COVID-19 pandemic. Unfortunately, information about admission to ICU and mechanical ventilation were not available at the SIVEP-Gripe form and, therefore, we could not assess whether they were more frequent among pregnant women or non-pregnant women. However, as mentioned before, since among notified cases pregnant women were more likely to be hospitalized, we hypothesized that actually they could have been prioritized to healthcare resources, especially during the COVID-19 pandemic, which affected a large part of the Brazilian population, overloaded the health system, and demanded the rationing of caregiving. Based on the burden that previous respiratory pandemias had caused to the pregnant population, it is possible that pregnant women have received some priority in the allocation of resources in situations of insufficient supply, reducing mortality in this group, and reinforcing the benefits of public health policies in order to protect this vulnerable population. Considering what is available in the literature comparing pregnant and non-pregnant women, it is possible that this prioritization policy may have protected pregnant women from worse outcomes, not only in H1N1 pandemic but also in the COVID-19 pandemic, given the knowledge acquired from previous respiratory pandemics and the scarcity of health system resources in face of the burden caused by the COVID-19 pandemic.

We also speculated that the difference in mortality among pregnant and non-pregnant women could be related with the profile of older women and women with more comorbidities in the non-pregnant group, factors associated with greater morbidity and mortality in both diseases. By balancing the groups regarding age and comorbidities, we could mitigate this confounding effect and the death rate remained lower among pregnant women, reinforcing our hypothesis that a potential prioritization of care may have been beneficial to this population.

This study is a population study, representative of the entire Brazilian territory, for both the H1N1 and the COVID-19 pandemics, reflecting the demographic and clinical aspects of women of childbearing age. In addition, we prioritized diagnostic assertiveness by considering only cases confirmed with PCR testing. This way, we guarantee that our results exclusively deal with infections by H1N1 and SARS-COV2 in the two pandemics and infections were not caused by other etiological agents.

Nonetheless, due to the rigorous criteria for the diagnosis, we had to exclude many of the reported cases of SARS, especially during the H1N1 pandemic, when PCR testing was scarcer. In addition, like so many populational studies, the quality and completeness of the database forms and the dynamic changes in the notification criteria end up incurring reporting biases. In addition, over time, the notification form has gone through modifications and, since our main objective was to compare both pandemics, we had to stick the analysis to the information that was available in the form during both pandemics. Relevant data, such as abdominal pain, taste change and loss of smell information, for example, were not present in the form during the H1N1 pandemic. In the same way, data about mechanical ventilation and intensive care admission were also not available at the time of H1N1 pandemic and could not be compared. Moreover, during the H1N1 pandemic, the notification form did not have the postpartum status field, and this group of women was reported and analyzed together with non-pregnant women. Nevertheless, in a recent Brazilian population analysis, during COVID-19 pandemic, puerperal women did not significantly differ from non-pregnant nor puerperal women regarding need for intensive care unit admission, invasive ventilatory support, or death, and, therefore, the bias from analyzing puerperal women along with non-pregnant women has possibly been minimized [32].

In both pandemics of H1N1 and COVID-19, among hospitalized subjects, pregnant women with SARS were less symptomatic and presented lower rates of death than non-pregnant women, and this protection effect was more marked during the COVID-19 pandemic. Possibly, the prioritization of this vulnerable group of women may have favored their outcomes and that must be taken into account when planning healthcare policies in the face of respiratory pandemics.

## Figures and Tables

**Table 1:** Epidemiological and clinical characteristics of hospitalized women of childbearing age with SARS during H1N1 (2009/2010) and COVID-19 (2020/2021) pandemics in Brazil.

	H1N1				COVID-19				
	Pregnant (*n* = 2190)	Non-Pregnant (*n* = 3698)	OR (95% CI)	*p*-Value	Pregnant (*n* = 5151)	Non-Pregnant (*n* = 59,463)	OR (95% CI)	*p*-Value	Breslow–Day *p*-Value
Age (years)	25.02 ±6.11	27.56 ±10.86		<0.001	30.05 ± 6.88	38.46 ± 8.14		<0.001	
Ethnicity *				<0.001 *				<0.001	
Asian	14/1950 (0.7%)	33/3408 (1.0%)			47/4209 (1.1%)	539/47,984 (1.1%)			
White	1251/1950 (64.2%)	2626/3408 (77.1%)			1849/4209 (43.9%)	26,447/47,984 (55.1%)			
Indigenous	2/1950 (0.1%)	16/3408 (0.5%)			16/4209 (0.4%)	104/47,984 (0.2%)			
Brown	532/1950 (27.3%)	589/3408 (17.3%)			2017/4209 (47.9%)	18,254/47,984 (38.0%)			
Black	151/1950 (7.7%)	144/3408 (4.2%)			280/4209 (6.7%)	2640/47,984 (5.5%)			
*Comorbidities*									
Chronic cardiac disease	31/1925 (1.6%)	133/3406 (3.9%)	0.40 (0.27–0.60)	<0.001	319/1705 (18.7%)	9552/22,304 (42.8%)	0.31 (0.27–0.35)	<0.001	0.199
Chronic respiratory disease	105/1918 (5.5%)	306/3412 (9.0%)	0.59 (0.47–0.74)	<0.001	229/1688 (13.6%)	3595/20,152 (17.8%)	0.72 (0.63–0.83)	<0.001	0.134
Chronic renal disease *	9/1921 (0.5%)	47/3402 (1.4%)	0.34 (0.16–0.699)	<0.001 *	32/1605 (2.0%)	1519/19,242 (7.9%)	0.24 (0.17–0.34)	<0.001	0.389
Chronic hematologic disease	12/1918 (0.6%)	22/3398 (0.6%)	0.97 (0.48–1.96)	1.000	25/1616 (1.5%)	536/19,055 (2.8%)	0.54 (0.36–0.81)	0.003	0.160
Immunosuppression	38/1924 (2.0%)	143/3406 (4.2%)	0.46 (0.32–0.66)	<0.001	50/1613 (3.1%)	1843/19,394 (9.5%)	0.30 (0.23–0.41)	<0.001	0.077
Change city to access health care	471/2177 (21.6%)	745/3667 (20.3%)	1.08 (0.95–1.23)	0.243	1879/5151 (36.5%)	17,108/59,458 (28.8%)	1.42 (1.34–1.51)	<0.001	<0.001
Previous Flu vaccination	83/1696 (4.9%)	264/3021 (8.7%)	0.54 (0.42–0.69)	<0.001	844/2322 (36.3%)	6393/28,155 (22.7%)	1.94 (1.78–2.12)	<0.001	<0.001

For age, mean age is shown ± standard deviation. To compare differences in age, *t*-Student test was performed; to compare distribution of ethnicity of patients, frequency of comorbidities, health care access and previous flu vaccination, chi-squared test was performed (except for ethnicity in H1N1 group and chronic renal disease in H1N1 group, when Fisher exact test was used and is denoted by (*). To compare the odds ratios (ORs) between the two pandemics, Breslow–Day test was used.

**Table 2:** Symptoms and outcomes of hospitalized childbearing-aged women with SARS during H1N1 (2009/2010) and COVID-19 (2020/2021) pandemics in Brazil.

	H1N1				COVID-19				
Symptoms	Pregnant (*n* = 2190)	Non-Pregnant (*n* = 3698)	OR (95% CI)	*p*-Value	Pregnant (*n* = 5151)	Non-Pregnant (*n* = 59,463)	OR (95% CI)	*p*-Value	Breslow–Day *p*-Value
Fever	2048/2168 (94.5%)	3557/3671 (96.9%)	0.55 (0.42–0.71)	<0.001	3076/4496 (68.4%)	37,842/52,494 (72.1%)	0.84 (0.79–0.90)	<0.001	0.002
Cough	2091/2172 (96.3%)	3538/3674 (96.3%)	0.99 (0.75–1.31)	1.000	3668/4671 (78.5%)	43,622/53,819 (81.1%)	0.85 (0.79–0.92)	<0.001	0.312
Dyspnea	1618/2132 (75.9%)	2972/3639 (81.7%)	0.71 (0.62–0.80)	<0.001	2946/4514 (65.3%)	42,214/53,232 (79.3%)	0.49 (0.46–0.52)	<0.001	<0.001
Sore throat	1027/2070 (49.6%)	1890/3551 (53.2%)	0.87 (0.78–0.96)	0.009	1084/3952 (27.4%)	13,765/45,084 (30.5%)	0.86 (0.80–0.92)	<0.001	0.925
Diarrhea	184/1998 (9.2%)	548/3466 (15.8%)	0.54 (0.45–0.64)	<0.001	549/3836 (14.3%)	10,106/44,245 (22.8%)	0.56 (0.51–0.62)	<0.001	0.668

To compare the prevalence of a given symptom or outcome, chi-squared test was used. To compare the odds ratio (OR) between the two pandemics, Breslow–Day test was used.

**Table 3:** Mortality rate of hospitalized childbearing-aged women with SARS during H1N1 (2009/2010) and COVID-19 (2020/2021) pandemics in Brazil.

	Pregnant	Non-Pregnant	OR (95% CI)	*p*-Value	Breslow–Day *p*-Value
H1N1	195/2001 (9.7%)	437/3461 (12.6%)	0.75 (0.62–0.89)	0.002	<0.001
COVID-19	445/4569 (9.7%)	9276/53,351 (17.4%)	0.51 (0.46–0.57)	<0.001	
Propensity Score Matching, balancing groups by age, cardiac, respiratory, renal and hematologic diseases and immunosuppression					
H1N1	525.4/5315.4 (9.9%)	664.2/5528.2 (12.0%)	0.80 (0.71–0.91)	<0.001	0.002
COVID-19	5804.3/48,473.6 (12.0%)	9941.2/58,130.1 (17.1%)	0.66 (0.64–0.68)	<0.001	

## Data Availability

The data and R codes that were used to support the findings of this study are available in GitHub repository at https://github.com/observatorioobstetrico/H1N1-flu-and-COVID-19-pandemics, accessed on 1 October 2021. These data were derived from the following resources available in the public domain: https://opendatasus.saude.gov.br/dataset/bd-srag-2009-a-2012, accessed on 1 October 2021, https://opendatasus.saude.gov.br/dataset/bd-srag-2020, accessed on 1 October 2021 and https://opendatasus.saude.gov.br/dataset/bd-srag-2021, accessed on 1 October 2021.
